# *swt-3* transcripts are expressed in Z1/Z4 during embryogenesis and in the adult spermatheca

**DOI:** 10.17912/W2ZT02

**Published:** 2017-07-20

**Authors:** Dustin Updike

**Affiliations:** 1 ​Mount Desert Island Biological Laboratory; Salisbury Cove ME, United States of America

**Figure 1.**
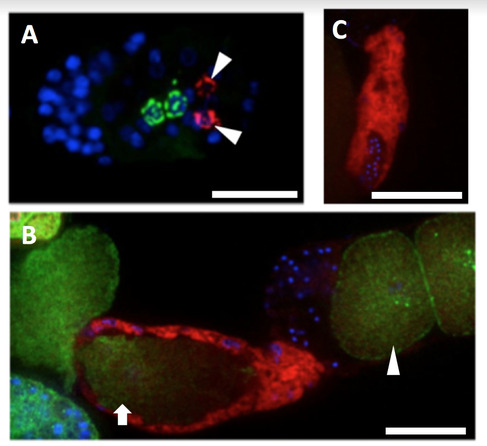


## Description

Stellaris smFISH probes targeting *swt-3*, an ortholog of the human solute carrier transporter *SLC50A1*, are shown in red (Cal Fluor 610), DAPI in blue, and PGL-1::GFP, a marker for P granules, in green. A) *swt-3* transcripts are expressed in Z1/Z4 cells during embryogenesis (arrowheads). The embryo shows Z1 and Z4 migrating to Z2/Z3 P granule cells (marked with PGL-1::GFP in green). B) A dissected proximal gonad of a young adult capturing the ovulation of a recently fertilized zygote (arrow) as it passes through the *swt-3* expressing spermatheca (red). Proximal oocyte marked with arrowhead. C) Sperm nuclei (blue) within the *swt-3* (red) spermatheca. scale = 20µ The experiment confirms and extends *swt-3* enrichment in cell-specific Z1/Z4 mRNA profiling experiment reported by Kroetz & Zarkower 2015.

## Reagents

The Stellaris *acdh-1* smFISH probe contains 44 oligos labeled with Cal Fluor 610 (sequences available upon request). Staining was performed as described by Ji and van Oudenaarden (2012). In short, PGL-1::GFP embryos and worms were fixed with formaldehyde and ethanol. Hybridization was done in the dark at 37°C for four hours. Cal Fluor 610 coupled probes were designed with the Stellaris Probe Designer from Biosearch Technologies.
